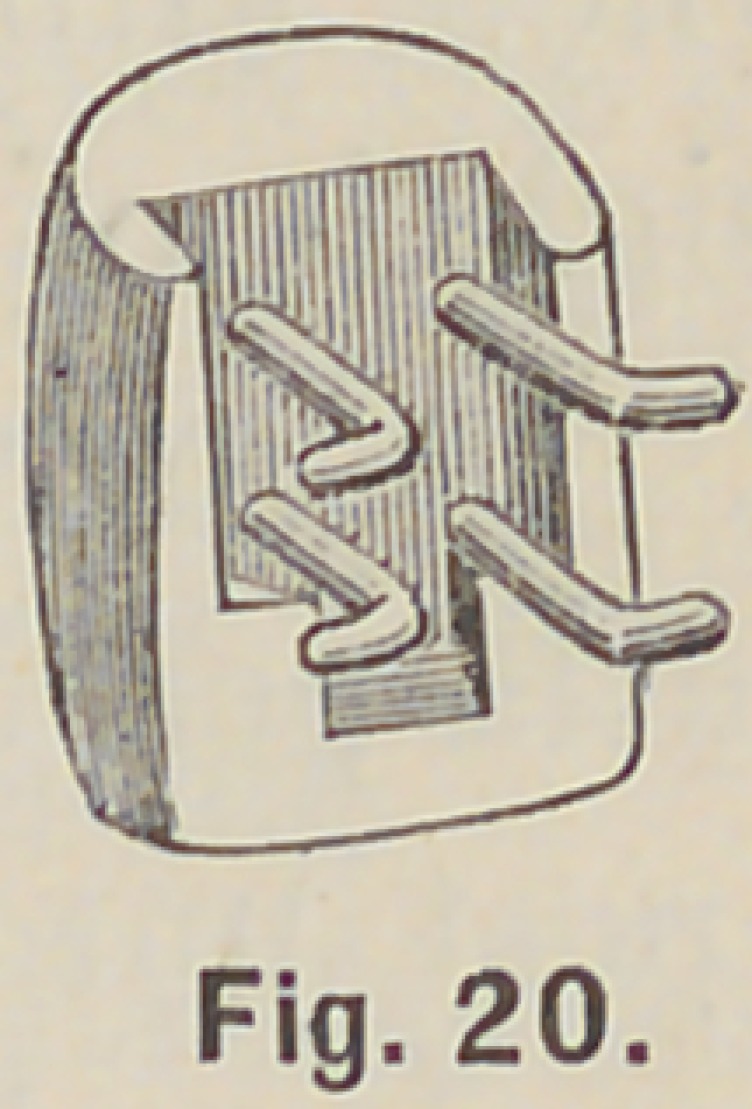# Description of a Novel Tooth-Crown

**Published:** 1883-07

**Authors:** W. Storer How


### Description of a Novel Tooth-Crown.

BY W. STORER HOW, D.D.S.

When the root is ready for mounting excise the decayed or broken crown, and cut the neck as usual for a pivot crown close up to the gum, using a barrel burr, No. 241, to cut the labial part of the root-neck above the margin of the gum without wounding it. Put a flexible gauge on a fine canal plugger, Fig. 1, and gauge the exact length of the canal from apex to neck. Then fill the canal for a short distance, and the gauge remaining in its first position will show how far from the apex the

Fig. 3.

Fig. 4.

Fig. 5.

Fig. 6.

Fig. 7.

canal is filled. Put another flexible gauge on a gates drill, Fig. 2, that should project from its gauge one-half the measured length of the canal, which is then drilled to the gauge on the gates drill. Set the twist drill in its chuck, Fig. 3, to project the same length as the gates drill, and drill the root to that depth. Set the tap in its chuck, Fig. 4, to a very little less length than the projecting twist drill, and putting oil on the tap, carefully tap the root to the gauged depth. Insert the screw post in its chuck, Fig. 5, until the post projects exactly the same length as the tap, and turn the thumb screw hard down on the enclosed end of the post. Take a square fissure burr, No. 59, and enlarge the hole in the root

Fig. 1.

Fig. 2.

one sixteenth inch deep all around to near the root margin, and with an oval burr, No. 94, cut a groove to further enlarge and under-cut the root opening, at each side and at the lingual part of the recess, Fig. 6, and be careful that no chips or moisture shall be left in the recess or canal. Screw the post into the root firmly yet without much force. Loosen the thumb screw without turning the chuck, which may thus be unscrewed a half turn and the post bent into the center line with the teeth, when the chuck may be unscrewed from the post, which if too long may be excised and ground off to let the teeth occlude. Now

Fig. 8.

Fig. 9.

Fig. 10.

Fig. 11.

Fig. 12.

Fig. 13.

try on the crown, Fig. 7, and if too thick at the neck, take engine disk, F, and without water, grind between the neck pins a little from the rib until the crown when pushed against the post is flush with the labial part of the root neck. Place the crown between the jaws of the spring mandril, Fig. 8, which will occupy the place of the post between the pins, and with the special pliers, Fig. 9, bend first the pin nearest the cutting edge carefully down on the mandril, Fig. 10, then bend the next opposite pin close to the first pin toward the crown neck, Fig. 11, and the other pins in the same order, excising them if too long to be bent close to the size of the mandril. Slip the crown over the post and try occlusion to see if the post is to be bent in or out, and then with engine stump-corrundun, No. 3, dry, grind the neck of the crown to fit the neck of the root closely and only at the labial part just under the gum, Fig. 12. Now grind the cutting edge to suit the other teeth, taking care that the opposing tooth does not strike either crown or post or pins ; then with the special pliers pinch the pins into the screw threads of the post until the crown is fixed on the root and perfectly adjusted in

Fig. 14.

Fig. 15.

Fig. 20.

Fig. 16.

Fig. 17.

Fig. 18.

Fig. 19.

Fig. 20.

relation to the teeth. Pack gold or amalgam or cement or Wood's metal thoroughly in behind the post—around the pins—between crown and root ; around the pins until at last the backing is rounded out for contour, Fig. 13, and the edges of the root and crown finished flush and smooth.

Fig. 14 shows a cuspid root with its crown, and also a bicuspid root and its crown, the lingual cusp normally present, and its crown ready to be slipped over the post as shown in Fig. 15, when the appropriate backing will complete the operation. The posts are made of a special metal with great exactness, and in a peculiar form, shown enlarged in Fig. 16, to ensure a firm hold on both the root and crown.

When drilling the bicuspid out notice the space between the drill and the natural cusp, and if it is not more than equal to the thickness of the drill the cusp should be cut vertically with a burr, so that when the post has been screwed in the crown pins will freely pass between the post and cusp. If a lingual cusp or shoulder is to be built, the pins may be twisted together over the mandril so that when in place on the post, they may be further twisted tight over the post, and the projecting ends, Fig. 17, serve as supports on which to build the cusp; and in some cases one pair of pins may be twisted and the other pair be bent as in Fig. 18. In every case the backing should, with very small points, be packed into all the crevices between the root and crown, and post and pins; and when a plastic backing is employed, there should always be a subsequent sitting for a careful, and thorough finishing of the joinings of the backing with the crown and the root. Roots that are very much wasted, may be drilled and tapped for a post which, as in Fig. 19, will hold the crown in place while the backing is being packed into the root, and around the post and pins; a root can thus be crowned, although a wide space may intervene to the extent of an eighth of an inch or more between the crown-neck and the root. A crown may be backed temporarily with wax, rubber, or gutta-percha, and at the next sitting the crown be taken off, the post unscrewed, and the root treatment continued until by repeated replacements the root is made ready for a permanent crown; or, a tubular threaded post may be employed, and the crown at once fixed; taking the precaution to insert a plug of wood in the tube, and after backing remove the plug, the ends of the crown-pins may be bent and the crown mounted on a rubber, or celluloid plate, as in Fig. 20.

The several applications were devised to afford facility and precision in doing the work, and it is believed that they will come into general use; but the crowns can be securely and quickly mounted on roughened or notched posts by any of the familiar methods; it is however only fair to expect that the merits, or defects of this crown will not be passed upon until it has been employed in exact accordance with the methods prescribed. For example, it may be supposed that almost any dentist can make a tap, and cut a suitable screw-post, but the fact is that it has taken a long time and many trials, often consultations with superior mechanics in Philadelphia, Providence, and Waltham, to produce the pecular and uniformly exact post and tap especially adapted to this purpose; the profession is therefore respectfully requested to test the crown by adopting the best methods.

## Figures and Tables

**Fig. 1. f1:**
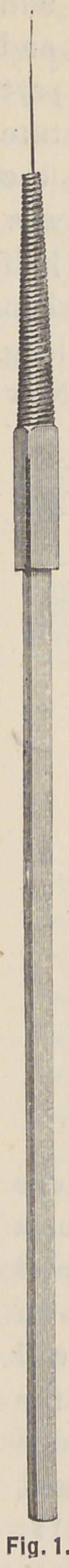


**Fig. 2. f2:**
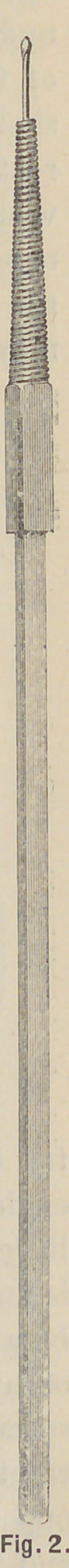


**Fig. 3. f3:**
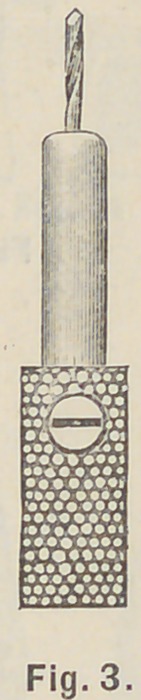


**Fig. 4. f4:**
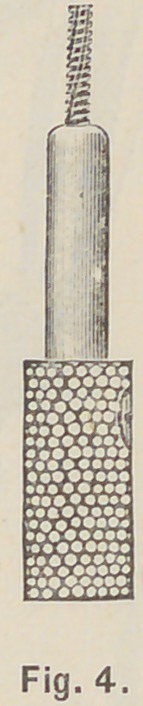


**Fig. 5. f5:**
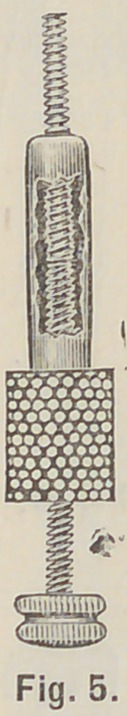


**Fig. 6. f6:**
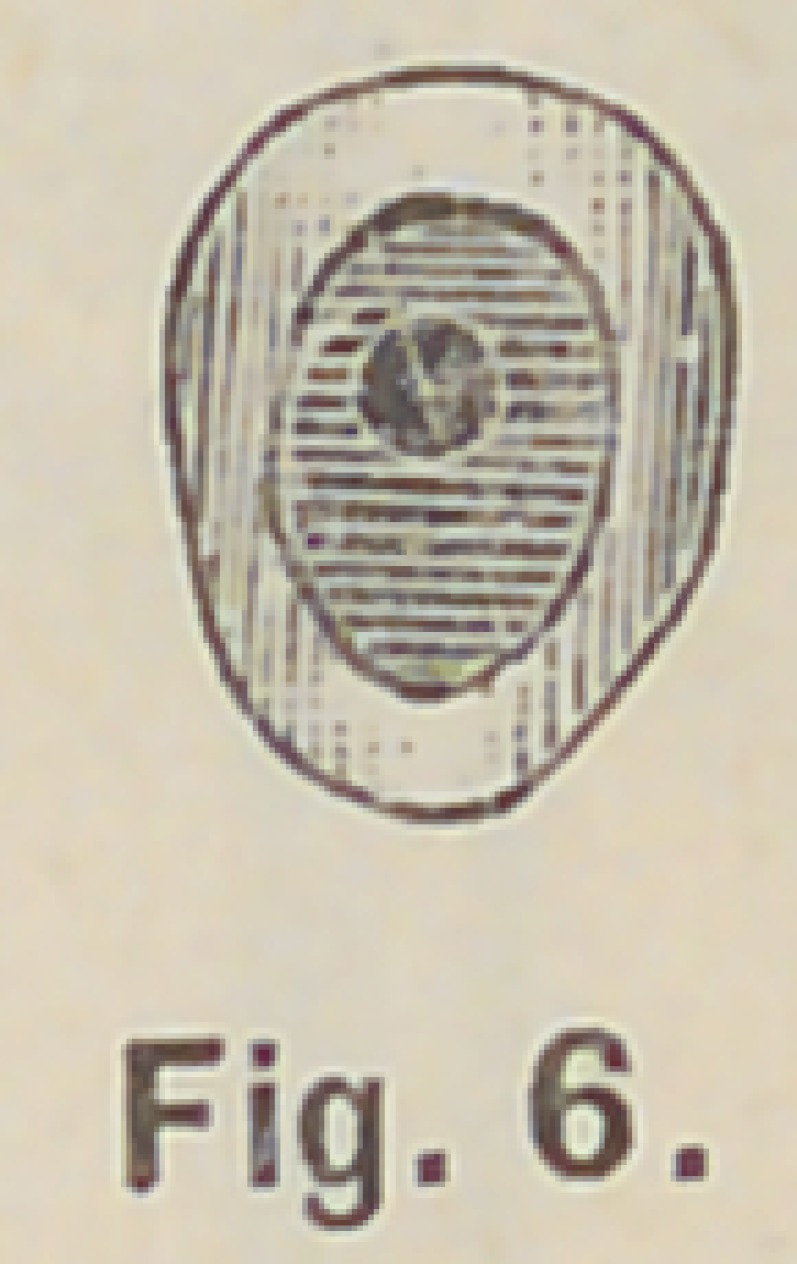


**Fig. 7. f7:**
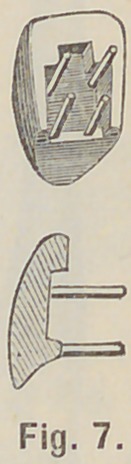


**Fig. 8. f8:**
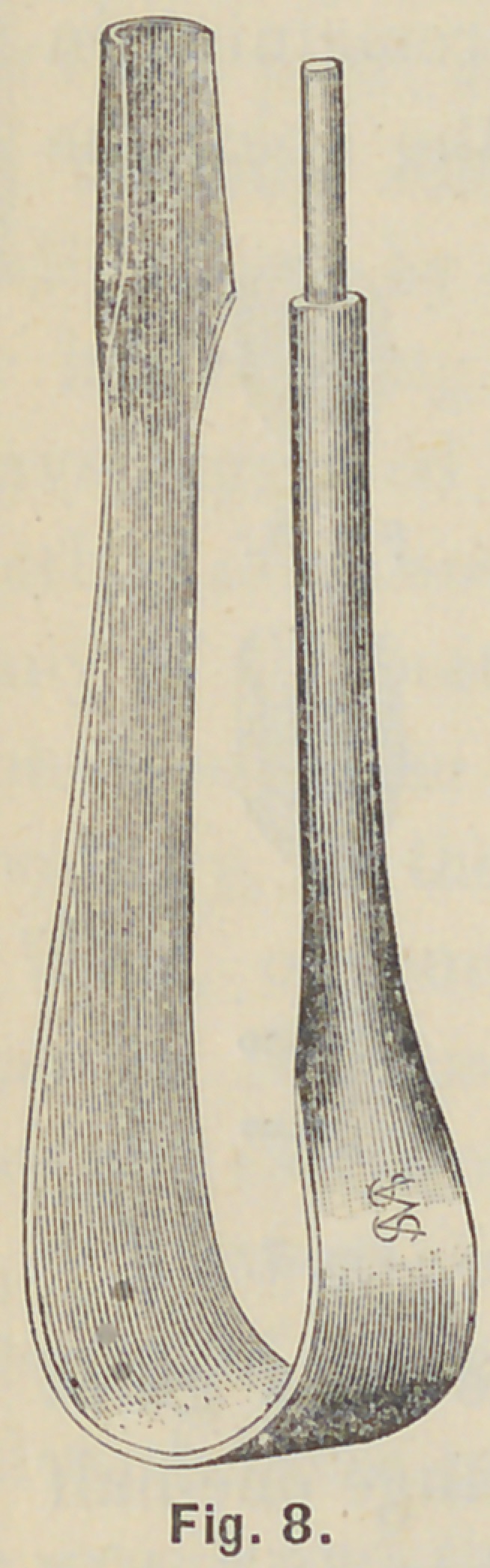


**Fig. 9. f9:**
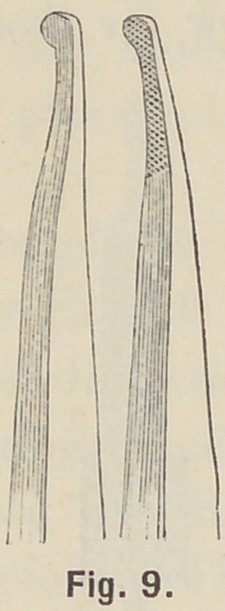


**Fig. 10. f10:**
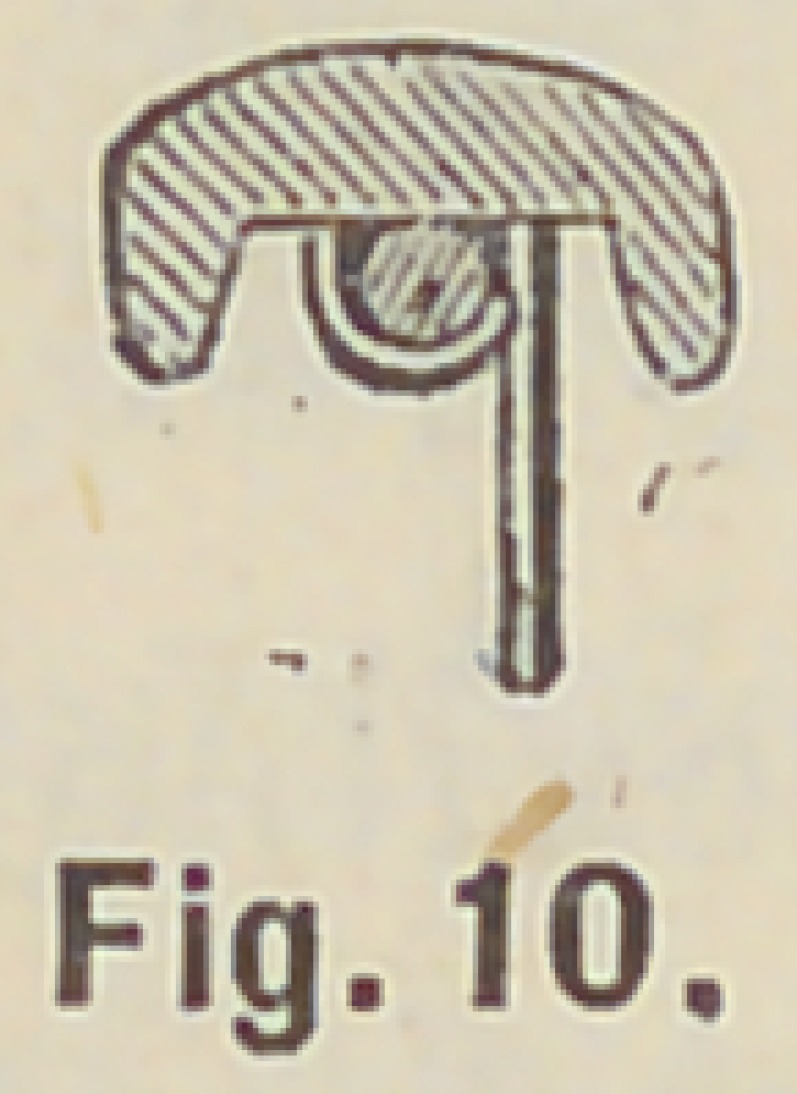


**Fig. 11. f11:**
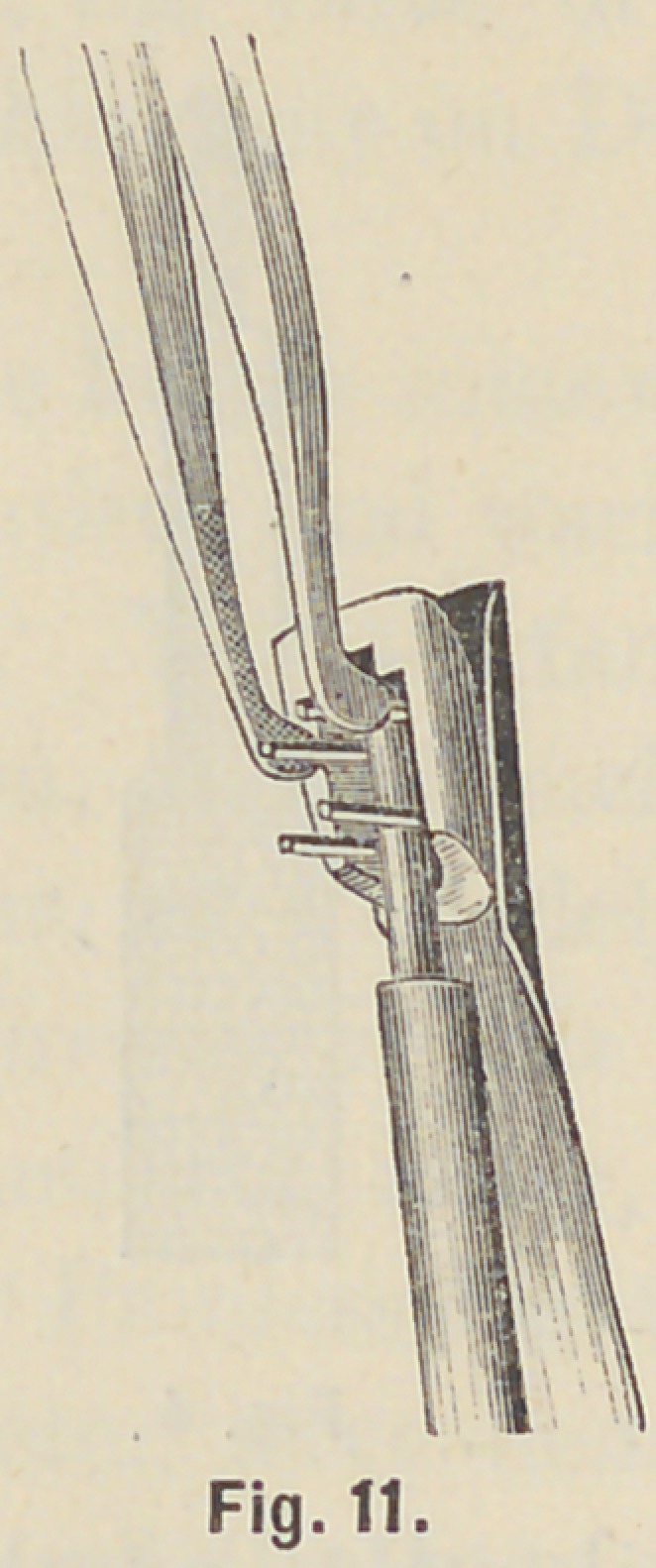


**Fig. 12. f12:**
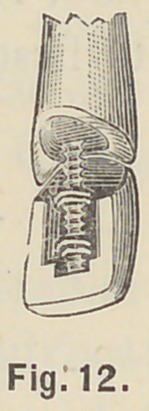


**Fig. 13. f13:**
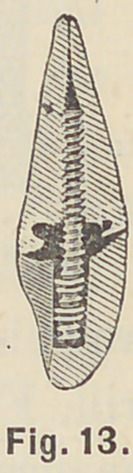


**Fig. 14. f14:**
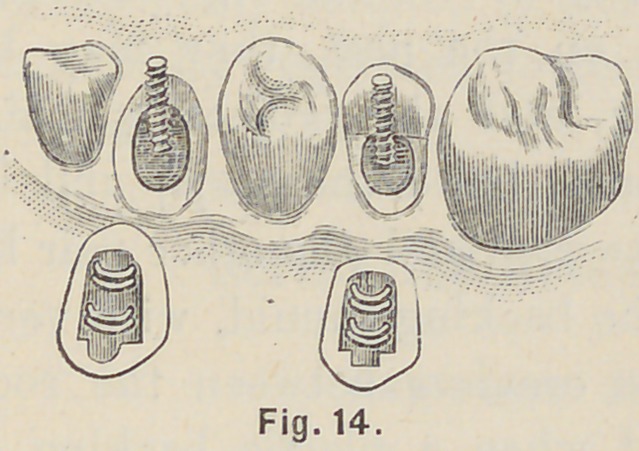


**Fig. 15. f15:**
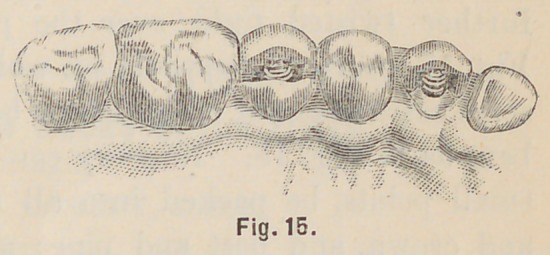


**Fig. 16. f16:**
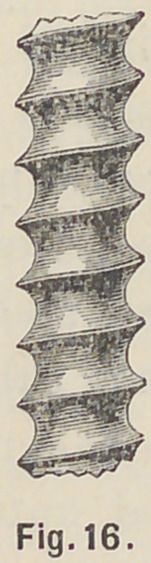


**Fig. 17. f17:**
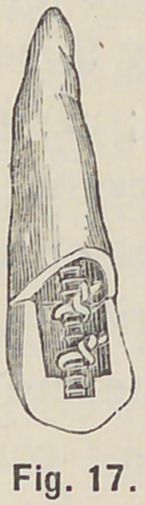


**Fig. 18. f18:**
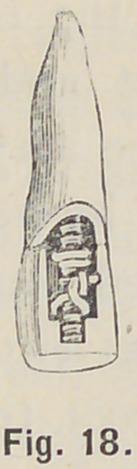


**Fig. 19. f19:**
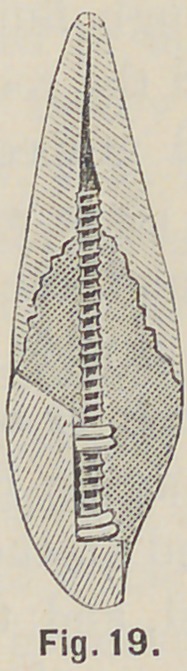


**Fig. 20. f20:**
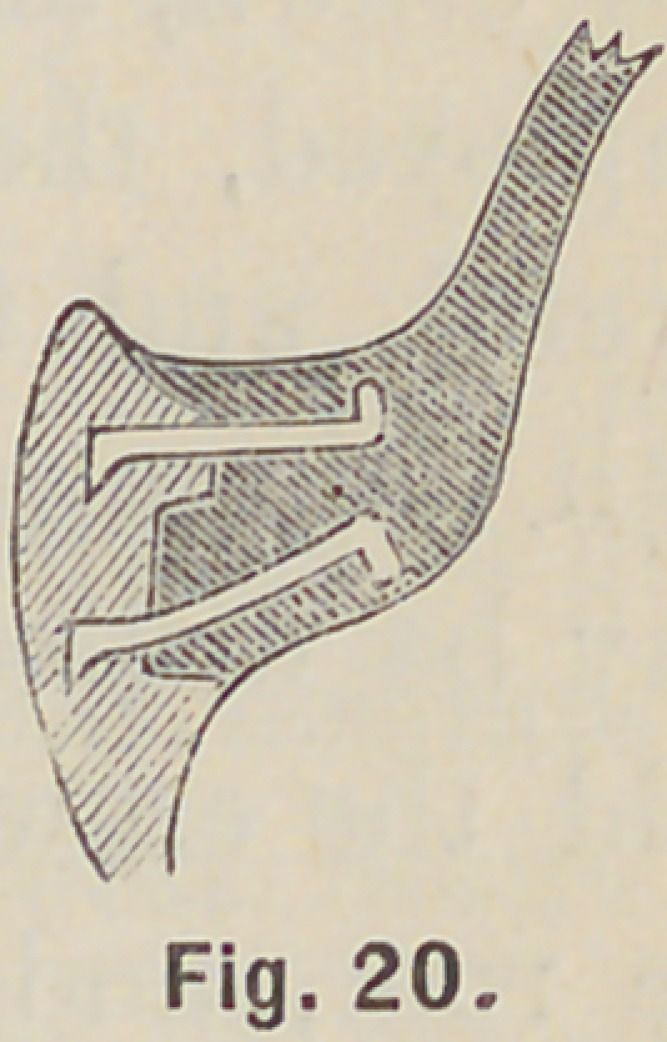


**Fig. 20. f21:**